# Maxillary Sinus Kaposi Sarcoma: Case Report in an HIV-Negative Patient with Thymoma

**DOI:** 10.1155/2017/3263728

**Published:** 2017-10-24

**Authors:** Bernardo Carvalho Araújo, Sara Viana Baptista, Luís Mascarenhas, Ezequiel Barros

**Affiliations:** ^1^Centro Hospitalar de Lisboa Central, Otorhinolaryngology Department, Lisboa, Portugal; ^2^Centro Hospitalar de Lisboa Central, Anatomical Pathology Department, Lisboa, Portugal

## Abstract

**Introduction:**

Kaposi sarcoma is an angioproliferative disorder that requires infection with human herpesvirus 8 (HHV-8) for its development. The majority of cases are associated with HIV infection or other immunocompromising conditions. Thymomas are occasionally associated to cytopenia, which may alter the patients' immune responses.

**Methods:**

Case report using clinical records.

**Results:**

Case report of a 46-year-old male patient diagnosed with thymoma and myasthenia gravis. The patient was referred to an otolaryngology consultation with complaints of facial pain in the right malar region, interpreted as an acute sinusitis. Following examination, an expansive maxillary sinus mass was found, and endoscopic surgery was undertaken. After careful investigation, it was diagnosed as a Kaposi sarcoma.

**Conclusions:**

It is thought to be the first described case of a maxillary sinus Kaposi sarcoma in an HIV-negative patient. Thus, this entity has to be considered in the differential diagnosis of sinus masses, even in non-HIV patients.

## 1. Introduction

Thymic neoplasms are responsible for 0.2 to 1.5% of total tumor incidence and are the most common neoplasms arising in the anterior mediastinum in adults [1]. They include both thymomas (20% of mediastinal neoplasms) and thymic carcinomas. Most thymoma patients are between 40 and 60 years of age, and there is a slight male predominance.

There are no known risk factors, and there is a strong association with paraneoplastic syndromes, the most common being myasthenia gravis (MG). MG has been diagnosed in 30 to 40 percent of thymic neoplasms; other paraneoplastic syndromes are rare [2]. MG is a neuromuscular junction disorder caused by an autoimmune reaction to the acetylcholine receptors of striated muscle; symptoms include diplopia, ptosis, dysphagia, weakness, and fatigue. Up to 50% of patients with thymoma have symptoms consistent with MG, which is common in all types of thymoma but is rare in thymic carcinoma; there is no sex predominance. Patients with thymoma and MG usually present with less advanced disease than those without MG, possibly because neuromuscular symptoms lead to an earlier diagnosis. Thymectomy usually results in reduction of the severity of myasthenia, although symptoms may persist in most patients.

Kaposi sarcoma (KS) is an angioproliferative disorder that requires infection with human herpesvirus 8 (HHV-8) for its development. As many as 4–15% of the healthy population may be seropositive for HHV-8; however, only under certain circumstances does KS develop, namely, in immunocompromised patients. KS is classified into four types based on the clinical ground in which it develops: (1) classic (the type originally described by Kaposi, affecting the oral cavity and skin, typically presenting in middle- or old-aged patients); (2) endemic (several forms described in sub-Saharan indigenous Africans prior to AIDS epidemic); (3) iatrogenic (associated with immunosuppressive drug therapy, usually diagnosed in renal allograft recipients); and (4) AIDS-associated (epidemic KS) [3].

In recent literature, some cases of association between KS and thymoma have been reported, most of them with cytopenia secondary to the thymic neoplasm (either pure red cell aplasia, erythroblastopenia, CD4+ lymphopenia, or Good syndrome—combined B and T cell immunodeficiency). Some reported cases include KS in HIV-negative patients, some in unique presentation sites [4–8].

## 2. Case Report

We report the case of a male patient aged 46. The patient had been diagnosed with MG at age 26, when symptoms of facial and limb extremities weakness were noticed for the first time. Following investigation, a thymoma was found as the cause of MG, and at age 32, he was submitted to a thymectomy and treated with acetylcholinesterase inhibitors and immunosuppressants, namely, corticosteroids.

**Figure 1:**
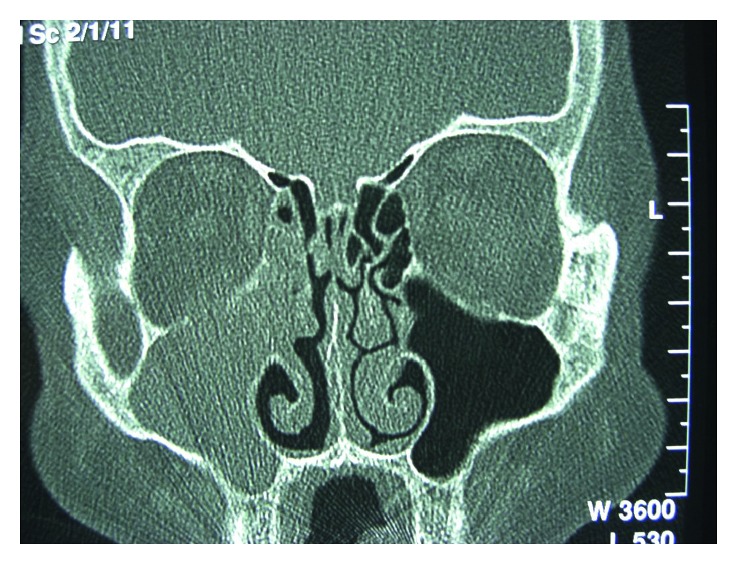
CT scan revealing an expansive mass occupying the right maxillary sinus (coronal view).

**Figure 2:**
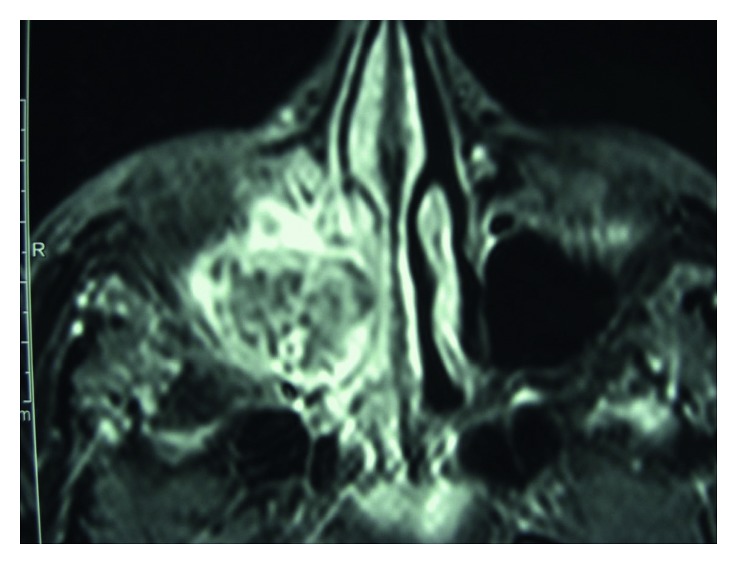
Magnetic resonance of the maxillary sinus mass.

At the age of 45, the patient was referred for the first time with complaints of facial pain in the right malar region and purulent nasal discharge from the same side. He was clinically diagnosed with acute sinusitis and underwent medical treatment, with slight relief of symptoms. As symptoms persisted after medical management, a CT scan was ordered, revealing an expansive maxillary sinus opacification with erosion of the right maxillary sinus medial wall (Figure 1()). The magnetic resonance imaging (MRI) revealed a mass in the right maxillary sinus with an heterogeneous pattern in the T1 sequence with contrast (Figure 2()).

**Figure 3:**
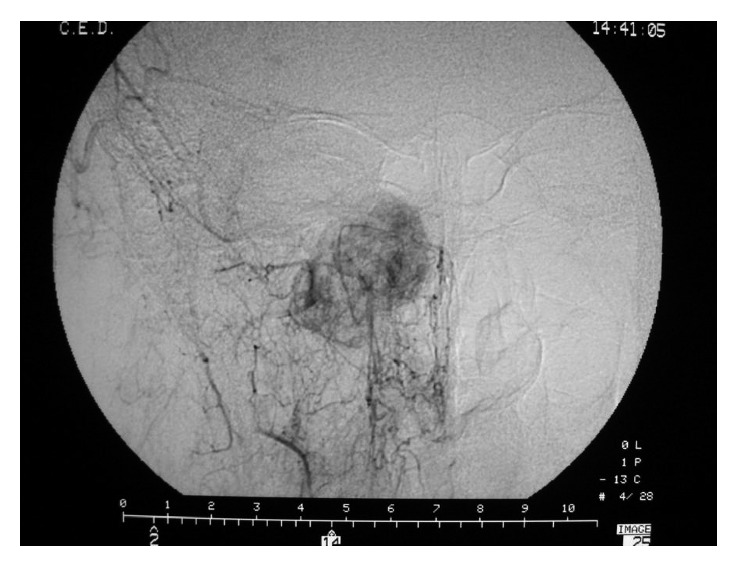
Angiography preembolization.

**Figure 4:**
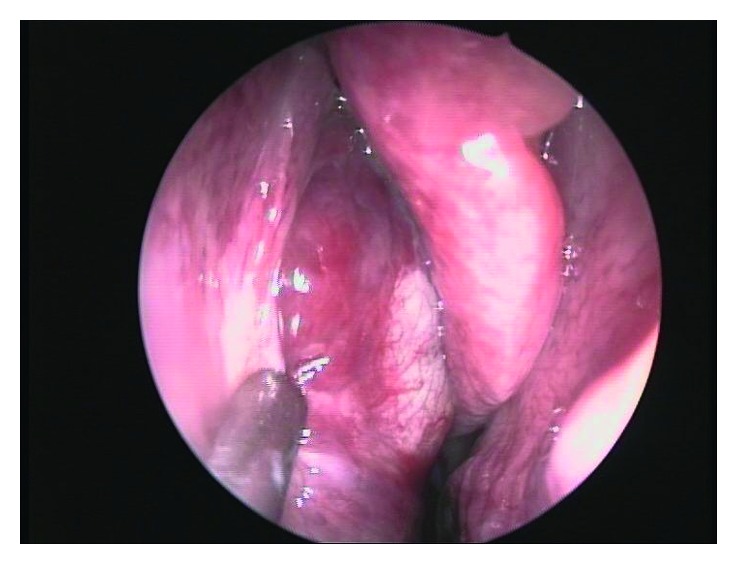
Endoscopic view of the mass.

An endoscopy for biopsy purposes was performed, but it was inconclusive due to clinically relevant hemorrhage from a vascularized lesion protruding in the medial wall of the maxillary sinus. An angiography and preoperative embolization (Figure 3()) of the right sphenopalatine artery was undertaken, and a meatotomy and ethmoidectomy under endoscopic control were performed (Figures 4() and 5()). Unexpectedly, the histologic result was a kaposiform hemangioendothelioma, a proliferation of endothelial fusiform cells, considered to be nearly exclusive of children and teenagers.

The patient remained stable with only symptomatic treatment until the age of 50 when, following worsening of MG symptoms (specifically astenia and fatigue), metastatic lung deposits of the thymoma were diagnosed. Surgical removal of the metastasis was undertaken.

The thymoma was classified as a B3 (WHO, 1999), and therefore, the patient was also treated with adjuvant radiotherapy. During radiotherapy and due to worsening of MG symptoms, human immunoglobulin and corticosteroids were administered.

Three months after these events, the patient had a new relapse from his MG and started corticotherapy. Two weeks into the treatment, he referred to newly skin pigmentation on the left wrist and legs. He also stated that these skin lesions were present 3 months earlier but smaller in size, growing with the recently added immunosuppressant therapy. These lesions were biopsied, and the histologic result was Kaposi sarcoma (KS). The patient was found to be HHV-8 IgG positive and HIV 1/2 and HTLV I/II negative.

## 3. Discussion

It was the diagnosis of KS in the biopsy of the limbs' lesions that led us to rethink the previous finding of the maxillary sinus kaposiform hemangioendothelioma, since the latter is usually not associated with HHV-8 IgG positivity nor is seen in adults. An histological review of the operatory sample was performed, and with immunohistochemistry technique (not previously available in the hospital), it was rediagnosed as a KS of the maxillary sinus.

KS in paranasal sinuses has been reported extensively in the literature, however, always associated with HIV infection or with immunosuppressant therapy. KS associated with hematologic, lymphoid, and thymic neoplasia has also been described, but no cases have been reported in the paranasal sinuses [9]. The KS in the extremities was most probably associated with the immunosuppressant therapy, since it developed right after the beginning of corticotherapy.

Extensive research on the characteristics of the paranasal sinus histology was undertaken, in order to explain the propensity of the maxillary sinus to develop KS (in both HIV-positive and HIV-negative patients) compared to the other paranasal cavities. No differences between maxillary sinus and the other paranasal sinuses were found to be significant in the literature to explain this propensity, and therefore it is still an open subject for further investigation.

## 4. Conclusion

Using the words “kaposi,” “sarcoma,” “maxillary sinus,” “HIV,” and “HIV negative” in different combinations in the databases PubMed/MEDLINE and Cochrane Database of Systematic Reviews (Issue 4 of 12, April 2014), there were no findings of cases in the recent literature of a maxillary sinus KS in an HIV-negative patient. Therefore, the authors here present what is thought to be the first described case of a maxillary sinus KS in an HIV-negative patient. We alert for care in the differential diagnosis of paranasal sinus lesions if concomitant with a thymoma, because the immunosuppressive treatment of the second, or the disease itself, can cause the development of the KS, regardless of an associated HIV infection.

There is still the need to keep investigating the specific features of the maxillary sinus that lead it to be more susceptible to these lesions.

## Figures and Tables

**Figure 5:**
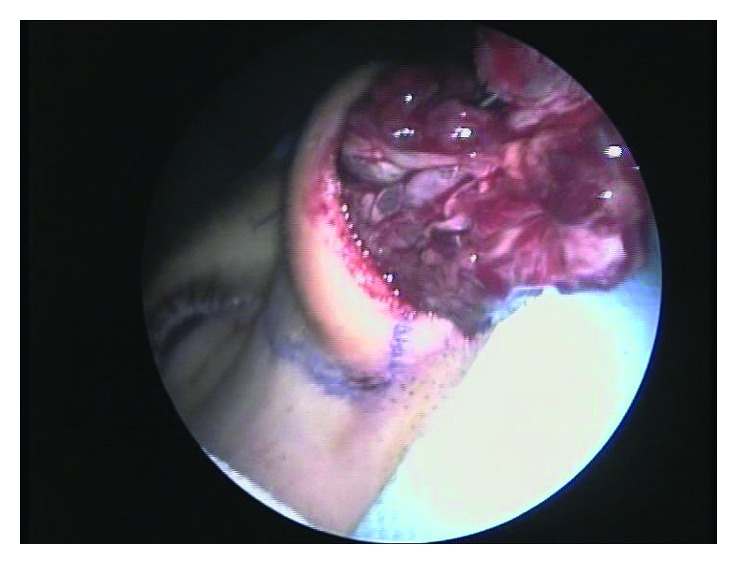
En bloc excision of the mass.
